# Comparative Genetics of Canine and Human Cancers

**DOI:** 10.3390/vetsci12090875

**Published:** 2025-09-10

**Authors:** Richard Curtis Bird, Bruce F. Smith

**Affiliations:** 1 Department of Pathobiology, College of Veterinary Medicine, Auburn University, Auburn, AL 36849, USA; 2 Scott-Ritchey Research Center and Department of Pathobiology, College of Veterinary Medicine, Auburn University, Auburn, AL 36849, USA; smithbf@auburn.edu

**Keywords:** canine, human, cancer, genes, therapy, model system, spontaneous disease

## Abstract

Domestic dogs develop cancer at rates similar to, or greater than, humans and suffer from cancer types that also reflect several similar common types of human disease. Canine cancers have, as a consequence, been investigated as a model of human cancer that is particularly attractive because it is an intermediate sized animal model with an intact immune system that develops spontaneous disease. This discipline has had extraordinary consequences for canine medicine as well, promoting the exploration of the genetics of canine cancers and the development of new therapies designed to be translated to humans but that also benefit the dogs themselves. We review the gene defects found in the most well-studied canine cancers and the new therapeutic approaches such investigations have promoted.

## Simple Summary

Dogs and humans both frequently develop cancer as they age. Because dogs share our environmental risks and exposures, they also provide a special opportunity to study cancer in an accelerated model due to their much shorter lifespan. Dogs provide an intermediate-sized animal model of spontaneous malignancy, with an intact immune system, that shares a remarkable similarity in terms of genetic defects and natural history. We review these aspects of canine cancer and their use in the development of innovative therapeutic strategies that can benefit both canine and human patients.

## 1. Introduction

Humans are considered among the most diverse of species on the planet, encompassing extraordinary genetic diversity, particularly when considering the routes of our evolution and worldwide dispersal starting from eastern and southern Africa [1,2]. Perhaps surprisingly, domestic dogs represent an almost equally diverse species probably because they accompanied early humans as we populated the globe. Most likely originally derived from domestication of an ancient wolf species, such as the Asian wolf as much or more than 17,000 years ago and perhaps much earlier, dogs have diversified into an extraordinary array of breeds [3,4,5,6]. Although domestic dogs represent enormous diversity they also, somewhat paradoxically, represent examples of extreme inbreeding (line breeding) within individual breeds where founder effects can frequently be profound [4,5,6]. Their well-defined genetics, in combination with the high incident frequency and the variety of canine cancer types represented, has supported speculation that dogs could provide an improved intermediate model for the development of new therapeutic strategies for cancer treatment. Dogs have also been proposed as sentinel indicators of human health because they share our lived environment—our shared exposome and its intimate relationship to cancer risk [7]. Such intermediate immune-intact models of spontaneous disease are especially important with the development of new immunotherapies. For example, recent clinical trials are being evaluated where tumor infiltrating lymphocytes, propagated and modified in culture, are reinfused into human patients [8]. Dogs can provide a promising preclinical model of intermediate scope for the development of such new and innovative therapeutic strategies [9].

## 2. Genetics of Canine Cancer

### 2.1. How Similar Are Domestic Dogs to Humans in Terms of Cancer Genetics?

Recent data suggests dogs develop cancer at rates similar to humans, depending on the tumor type, and canine rates may rise to perhaps as high as 5-fold more frequently than humans in some cases. A wealth of published data has confirmed that domestic dogs develop their cancers due to defects in many of the same genes found defective in human cancers [10,11]. The genes can be divided into two genetic groups—gain of function oncogenes and loss of function tumor suppressor genes [12,13,14]. And, at least where it has been investigated (Table 1), the number and types of defects or mutations appear similar to those identified in human cancers [12,13,14]. This includes canonical protein encoding genes, associated with phenotype, as well as non-coding genes that give rise to functional RNAs such as microRNAs [13,14,15,16,17].

### 2.2. How Similar Are Domestic and Wild Canids in Terms of Cancer Genetics?

Recent sequence analysis has confirmed that domestic dogs originally developed from wolves [4,6]. As a consequence, interest in wolf populations and their cancer risk has also been the subject of investigative interest. Wild and domestic canids are very similar in terms of their genetics and are still capable of crossbreeding successfully [18]. Domestic dogs are considered a subspecies (*Canis lupus familiaris*) of wolves (*Canis lupus*) [18]. This similarity has complicated assessment of the domestication of dogs due to difficulties in calculating divergence due to continued gene flow between wild and domestic populations [19,20,21,22,23].

It is not surprising that cancer is rarely, if ever, found in wild populations, most likely due to much reduced lifespans in the wild and difficulty in timely identification of samples. Lifespan for wolves in the wild probably averages about 3–5 years [24,25,26]. In captivity, cancer is thought to be responsible for a large percentage of deaths of captive wolves [24,25,26]. Because solid tumors in domestic dogs are most frequently geriatric diseases, it is unlikely wolves in the wild would be old enough to develop cancer. Once managed in a captive environment, however, wolves develop cancers frequently and this is the most common cause of death approaching 50%. The enhanced cancer frequency may be due to the same genetic predispositions found in domestic dogs and only becomes evident once wild canids are able to live much longer [24,25,26]. This strongly suggests that canine predisposition to cancer is an inherent trait and, like human disease, the result of gene variants that increase cancer risk. Recent evidence has identified potential inherited cancer predisposition, such as canine alleles of the CEACAM gene for example, that are associated with specific tumors and breeds [27,28,29]. However, no canine risk variants have yet been validated, and currently, the majority of disease appears to be spontaneous and due to somatic mutation [27,28,29].

Domestic dogs live, on average, to 12 years old for all breeds with larger breeds having shorter average lifespans and small breeds having longer lifespans [30]. Cancer in domestic dogs most frequently appears after 5 years of age and especially after 8 years of age. Domesticated dog breeds that appear particularly susceptible to cancer, such as boxers, average approximately 20–30 kg (44–66 lb) and develop cancers frequently enough to shorten their lifespan by approximately 3–4 years compared to other breeds of comparable size [30]. Boxers were the first canine breed to have their genomic completely sequenced as it was anticipated that there would be identifiable tumor suppressor defects found, although to date this has not been the case (E. Ostrander personal communication).

### 2.3. Are There Associations Between Individual Breeds and Individual Cancer Types in Domestic Dogs?

There have been many attempts to associate particular cancer types with individual domestic canine breeds [31]. However, the consensus appears to be that, although there may be small biases detected for some breeds, the overarching conclusion seems to be that there is very little bias associating particular cancers with individual breeds. As a recent example, an analysis of the data from the Swiss Canine Cancer Registry from 2008 to 2020 reported on the ten most common canine tumors in the twenty most popular Swiss breeds [31]. A review of the data suggests that all of these popular breeds contract all of the ten most common cancers. And, although there are clearly some differences, the overwhelming impression is that there is a surprisingly uniform distribution of cancer types among all popular breeds [31]. What is most remarkable, however, are the differences in frequency of cancer generally among breeds. Four specific breeds are clearly much more frequently represented when assessing incident cancer rate. Those breeds include, from the most susceptible, Cocker Spaniel, Golden Retriever, Labrador Retriever and Bernese Mountain Dog. It should be noted that highly cancer-prone breeds, such as Boxers, were not included in the top 10 most popular Swiss breeds [31]. Care must also be taken in such studies, to account for large differences in population sizes between breeds in different regions.

## 3. Canine Cancer Types and Their Genetic Defects in Comparison to Human Disease

Like most other mammalian species, dogs can develop a wide array of cancer types [32,33]. However, there are a few more common cancers of dogs that have been investigated to reveal at least a portion of the genetic basis and defects associated with disease. This is because these canine cancers present the most common health threat to privately owned pet animals and because they also closely resemble similar cancers with comparable pathology in human patients [32,33]. These canine cancers have, as a consequence, been developed as effective spontaneous models of human cancer in order to exploit these similarities in the development of new therapeutic strategies. Earlier reviews of the potential for several canine cancers to become effective models of human disease have predicted the application of recent advances in cancer immunotherapies and other strategies to canine cancer treatment [34,35].

### 3.1. Canine Breast/Mammary Cancer

Canine breast/mammary cancers are among the most frequently occurring cancers in middle aged to geriatric unspayed female dogs with only small differences in occurrence between breeds or animals of different size and this has varied between reports [36,37,38,39,40]. Cocker Spaniels, Golden Retrievers, and boxers may, however, be at somewhat higher risk of mammary cancer [41]. The development of mammary cancer is divided into carcinomas and sarcomas originating from mammary tissue. Histological classification of mammary tissues including tubular phenotypes, as well as a mixed cancer phenotype [42]. The former (carcinoma, adenocarcinoma) have been shown to model carcinomas in human female patients quite closely, while the latter phenotype appears largely unique to dogs. Canine mammary carcinomas have been subtyped into luminal A, luminal B, HER-2 positive and possibly, triple-negative phenotypes using a gene expression profile similar to the subtyping applied to human disease and based on quantitative reverse transcriptase PCR [14].

Characterization of canine subtypes of mammary cancers has only recently been applied, and is still not commonly used (Table 2), despite the early identification of HER-2 positive expression in these tumors [43]. Despite this, recent data has suggested that the majority of canine cancers are Luminal A or Luminal B in phenotype (ER+/− or PR+/− but not both deleted and erbB-2/HER-2− for Luminal A and erbB-2/HER-2+ for Luminal B) with the most aggressive forms often being characterized as HER-2 positive (ER−, PR−, erbB-2/HER-2+) in canine patients [14,43]. There is a distinct absence of triple-negative tumors found in these populations although there have been reports of triple-negative canine mammary tumor cell lines in the literature [44].

In mammals and other animal species, the CDKN2A/B or INK4A/B locus encodes 2 key genes that regulate cell cycle entry and exit. Both the p16/INK4A and p14/ARF (alternative reading frame) transcripts are encoded by the same gene; however, they employ alternative first exons, identified as 1β (p14ARF) and 1α (p16/INK4A) [44]. Because the exon 1β coding region ends out of frame, compared to exon 1α, the remainder of the gene in exons 2 and 3 are read out of frame thus encoding completely different protein sequences. This unusual structure results in two different genes with completely independent functions co-evolving from the same sequence because they are read out of frame. This means there can be no third base wobble as any mutation will affect both sequences and at least one will encode a first or second codon nucleotide. Because p16 suppresses cell cycle G1 phase cyclin/CDK 4 or 6, which suppresses the Rb protein, and p14 suppresses MDM2, which suppresses p53 protein, this single gene locus regulates the two most important functional regulators of cell cycle exit [44]. Dysfunction of this single site, through mutation, can result in defects in both the Rb and p53 pathways thus rendering one of both pathways defective the two most important regulators providing a brake on cell proliferation and cell cycle progression. Ultimately, such mutations are highly oncogenic and common in many types of cancer in dogs and humans [44].

**Figure 1:**
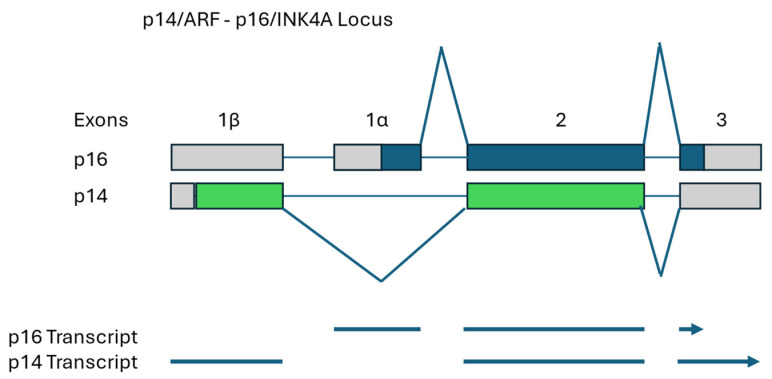
Mutations in Coevolving INK4A/p16 and p14/ARF Genes Inhibit Both p53 and Rb Pathways.

Analysis of the gene defects in these canine tumors has revealed some commonalities shared by a majority of the tumors. The most frequent defects are deletions of various sizes found in the INK4A/p16 (CDKN2A) gene and larger deletions frequently affecting neighboring INK4B/p15, INK4C/p18 and INK4D/p19 genes as well. Because this unique locus involves INK4A/p16, it also involves the p14ARF gene (alternative reading frame) encoded by an alternative first exon and subsequently read out of frame through exons 2 and 3 of the INK4A/p16 gene. This very unusual overlap of out of frame but overlapping reading frames results in two completely unrelated proteins—one that suppresses the G1-phase cyclin-dependent kinases 4/6 (INK4A/p16), and another, p14ARF, that suppresses the MDM2 ubiquitin ligase that inactivates p53 [44]. Thus, with one deletion/defect both the major tumor suppressor that blocks G1 phase progression, through inhibition of CDK 4/6 which inhibits, in turn, Rb (retinoblastoma) protein, and the major apoptotic inducer, that detects DNA damage and blocks cell cycle progression in all phases, p53 protein, are both inhibited (Figure 1).

The p16 protein open reading frame (dark blue) is encoded from the middle of exon 1α through the beginning of exon 3 while the p14 protein open reading frame (green) is encoded from near the beginning of exon 1β through to the end of exon 2 where a stop codon exists (Figure 1). The p14 transcript includes exon 3 sequences but they encode only the region (gray) encoding the untranslated 3′-tail. Although conserved for sequence, there are small differences in protein length between dogs and humans. Human p14 is 132 amino acids while canine p14 is 129 amino acids. Human p16 is 156 amino acids while canine p16 is 151 amino acids in length. This gene has been found to encode key dysfunctional mutations in many canine and human cancers and has been investigated primarily in mammary cancer and melanoma in dogs [44].

Other gene defects that affect expression have also been found in canine mammary cancers. These include the estrogen and progesterone receptor genes whose expression is strongly correlated with luminal A and luminal B phenotypes in dogs and humans and the HER-2 receptor (c-erbB-2) in those canine and human mammary cancers that are of the HER-2 positive and Luminal B phenotypes [14]. Additionally, strong risk associations have been identified in canine cancers that also include activations of expression in one or both of c-erbB-3 and c-erbB-4 genes [14]. Apart from these more canonical breast-cancer-associated genes, frequent defects have also been detected in p53 expression linking such defects to p14/ARF defects [14]. As a consequence, and because these defects are similar to spontaneous human breast cancers, canine mammary tumors have been suggested as promising models for the development of new anti-breast cancer therapeutic strategies [9].

A successful canine mammary tumor therapeutic vaccine has been reported in which a hybrid cell fusion between autologous dendritic cells from the patient and unmatched canine mammary tumor cells was employed to promote patient immunity [45,46]. Three applications of this construct, including gemcitabine treatment and CpG oligonucleotide adjuvants, were effective in treating canine mammary cancer. The patient animals treated had been surgically resected and had no evidence of metastatic spread. In each case, the patient animals survived for an average of 3.3-fold longer (median survival 611 days) than unvaccinated control patient animals (median survival 184 days) and also appeared to enhance quality of life as assessed by the owners (lack of evident pain, robust appetite, mobility, and overall demeaner). No vaccinated animals died as a consequence of mammary cancer. Vaccinated animals were followed until death from other causes or loss of contact. In only one case was detectable recurrence noted but this did not cause the death of the patient [46].

There have also been reports of the DNA repair associated tumor suppressor genes BRCA1 and BRCA2 and expression defects in canine mammary cancers [26,41]. Although they are well known as a source of hereditary predisposition in human cancers, less is known about their contributions to canine cancer. Germ line, mutations may be associated with higher cancer risk although distinct differences between human and canine expression profiles and those of accessory proteins are yet to be fully delineated.

### 3.2. Canine Malignant Melanoma

Canine malignant melanoma is among the more frequently diagnosed cancers in dogs, similar to humans [36]. Human melanoma is thought to be caused by environmental ultraviolet light exposure resulting in genomic mutations, with about half involving mutations of the BRAF and NRAS genes [37]. The majority of canine oral melanomas present as oral disease developing in the buccal tissues of the mouth [47]. Surgery is the standard of care but can complicate recovery [33]. Like human disease, it is possible to cure canine melanoma; however, in most cases this is not achieved and disease is often fatal.

BRAF gene mutations in human melanoma result in an activated oncogene that constitutively activates a pro-proliferation pathway for melanocytes that promotes oncogenesis in more than half of all human melanomas [48]. Canine melanoma, to date, does not seem to involve the BRAF pathway, although this only accounts for approximately half of human disease and canine melanoma has been suggested as a potential model as canine disease may more closely resemble the remaining half of human disease [37,49]. Despite these differences, there are well-characterized defects that have been identified. The c-kit gene has had mutations identified in canine patients by several authors [47,50,51]. Additionally, the HER-2 gene, well characterized in both breast and other hormone-dependent cancers, also appears to be activated in canine melanomas [52]. The p16/INK4A gene and the PTEN gene have also been identified as key mutation targets in canine melanoma [12]. The significant expression of PD-1/PDL1/PD-L2 molecules by both human and canine melanomas suggests exploitation of inhibitory strategies could potentiate the effects of TIL (tumor infiltrating lymphocyte) therapy on canine melanomas [53]. Finally, mismatch repair deficiency has been identified at high frequency in canine melanoma [54].

### 3.3. Canine Osteosarcoma

Canine osteosarcoma is another common tumor of dogs, and is the most common canine tumor of bone. Large breed dogs are affected at higher rates as are specific breeds, suggesting both physical and genetic predispositions are involved [55]. The disease resembles osteosarcoma in humans, with the exception that human patients are generally in their teens or twenties, while canine patients are mostly geriatric. Small peaks of incidence are seen in both younger dogs and older humans. Both humans and dogs have poor survival rates when the disease metastasizes and metastasis is common [56].

Initial attempts to understand the genetic defects underlying canine osteosarcoma resulted in the investigators finding differently expressed genes in tumors from each species. Additionally, heterogeneous chromosomal alterations were also seen in this tumor [55]. An early study showed four of five cell lines had elevated Akt levels, which is normally suppressed by PTEN, a tumor suppressor. This was determined to be due to an absence of PTEN expression in 3 of the lines and an inactivating mutation in the fourth. When primary tumors were examined, ten of fifteen samples also showed variable or no PTEN [57]. However, complicating interpretation of the importance of PTEN, is a later study on a much larger cohort of 95 canine osteosarcomas where PTEN reduction was only observed in 25% of the tumors [58]. In a separate study, activation of COL6A3, COL5A2, TNC, and ITGB5 genes were demonstrated. These are part of the PI3/Akt focal adhesion pathway and demonstrate the importance of this pathway to osteosarcoma [59].

Genetic risk factors have been implicated in the dog for many years due to the breed-related incidence of osteosarcoma. By grouping dogs by breed, gene expression profiles were derived that grouped dogs into two subgroups with respect to outcome. These profiles overlapped profiles known for soft-tissue sarcomas [30]. Genome-wide analysis has identified 33 loci as heritable risk factors for osteosarcoma. The strongest of these was CDKN2A/B (INK4A/B), which was identified in greyhound dogs. This locus was identified as fixed for the risk allele in Rottweilers and Irish Wolfhounds where additional loci were identified. This indicates that multiple loci are responsible for enhanced inherited risk of osteosarcoma [60]. Subsequent analysis has shown that a locus, most likely CDKN2A/B, on CFA11, was associated with osteosarcoma in Leonberger dogs [61]. GRB10 has been associated with osteosarcoma in Irish Wolfhounds and has also been linked to osteosarcoma in humans [62]. While mismatch repair deficiency is commonly identified as a risk factor in many tumors, and occurs with high frequency in canine melanoma and hepatocellular carcinoma, it was a rare finding in canine osteosarcoma, indicating that this pathway is not commonly dysregulated in osteosarcoma [54].

Deep sequencing has created a more refined picture of expression patterns in canine osteosarcoma. An exosomal gene expression signature in canine osteosarcoma identified five genes that were overexpressed, SKA2, NEU1, PAF1, PSMG2, and NOB1. These were validated by Q-RT-PCR. This signature, when detected post-therapy, was correlated with poorer survival times [63]. Subsequent bulk tumor sequencing on the transcriptomes of tumors from seven dogs with patient matched normal bone showed that, while gene expression profiles varied between tumors, they were more similar to each other than to normal bone [56]. This study also confirmed the heterogeneity of these tumors, as individual sample frequently showed significant difference in the up- or down-regulation of specific genes, when compared to the other samples. Subsequently, single-tumor single-nuclei multiome (ATAC + Gene Expression) sequencing has further demonstrated heterogeneity within tumor cells, identifying at least 3 separate clades of tumor cells within the tumor mass. Multiple components of the tumor microenvironment were also identified [64]. In another study, single cell RNA seq identified 10 tumor associated transcriptionally distinct cell types from six dogs prior to treatment. It was unclear from the publication how similar the gene expression profile in the identified tumor cells was between patients as the pool included fibroblastic (n = 1), chondroblastic (n = 1), and osteoblastic tumors (n = 4). Cross-species analysis did find a high degree of similarity with human osteosarcoma [65]. Gene expression patterns can be identified that correlate with prognostic value. These segregate canine osteosarcoma into poor and favorable prognoses. And, these gene expression signatures show similar prognostic results with human data sets; however, this does not apply once metastatic disease is present [66].

### 3.4. Canine Lymphoma

Canine lymphoma is a tumor of cells of lymphatic origin that predominantly affects the lymph nodes. It can be divided into subtypes based on the cell of origin of the tumor, such as B-cells, T-cells, or NK cells. Lymphoma is one of the most common cancer in dogs, and there appears to be an inherited component, as some breeds show an increased frequency of the disease. Most dogs that receive treatment go into remission; however, the disease usually returns at some point in the future and the chance of resistance to chemotherapy is increased with each relapse [67].

Early studies of canine lymphoma, using FISH (fluorescence in situ hybridization), showed shared and consistent cytogenetic abnormalities between canine and human hematopoietic tumors [68]. Genome-wide association studies on canine hemangiosarcoma and B-cell lymphoma in Golden Retrievers found two shared predisposing loci, located on chromosome 5. These loci were estimated to contribute 20% of the risk of each of these cancers. Genome sequencing of the identified areas found 3 shared haplotypes and one B-cell lymphoma specific haplotype. No coding differences were identified in risk haplotypes. Expression analysis of B-cell lymphomas with the first locus showed down regulation of several genes, including TRPC6, which is involved in T-cell activation. The second risk associated locus overlapped STX8, a gene related to vesicle transport and release [69].

Gene expression studies showed that the fragile histidine triad (FHIT) gene had reduced expression in canine lymphoma cell lines. It was unclear if CpG methylation was involved, as it is with human tumors. The gene was found to be deleted in all five cell lines examined. Due to the similarity of this defect with human tumors, it was hypothesized the FHIT may be an important gene involved in promoting the formation or growth of canine lymphoma [70]. Both T-cell and B-cell lymphomas expressed elevated levels of EP4R, a gene that is thought to protect lymphosarcoma cells from apoptosis [71]. Upregulation of Flt3 and VEGFA have been identified in acute lymphoblastic lymphoma [72,73]. Canine T-cell lymphomas have been shown to have lowered expression of SYK and KIT, while demonstrating upregulated MMP9 and TIMP1 expression [73,74,75].

When B- and T-cell lymphomas from dogs were characterized by transcriptomic analysis, along with melanoma, osteosarcoma, and pulmonary carcinoma, each tumor was found to have a unique cluster of differentially expressed genes, allowing all five tumor types to be individually classified. In addition, these expression signatures were successfully used to classify similar human tumors. The expression signatures uniquely showed elevated ribosomal proteins (RPL8, RPS7, and RPLP0) in B-cell tumors, and increases in genes involved in epigenetic regulation (EDEM1, PTK2B, and JAK1) in T-cell tumors [57]. RNA seq of CD4+, CD8+, and CD4−CD8− peripheral T-cell lymphomas (PCTL) showed that while CD4+ tumors had a consistent gene expression profile that included upregulation of GATA3 and PI3K/AKT/mTOR signaling and downregulation of PTEN, CD8+ and CD4−CD8− tumors were more heterogeneous [76].

Understanding gene expression in therapeutic situations may help better understand treatment responses and failures. Patients with relapsing T-cell lymphoma during the 1st round of CHOP-based treatment showed a unique RNA expression profile when compared to patients that successfully entered remission. This included downregulation of chemokine CC motif and CCL4 in the tumor, and upregulation of T-cell signaling genes, CD3E, ITK, and LAT [77].

In a study of 18 dogs with diffuse large B-cell lymphoma (DLBCL), the dogs were segregated into good and poor responders after chemotherapy. The investigators saw little similarity in differentially expressed genes between the two groups. Increased CCND3 was identified in the poor responders, while increased CREBBP, CDKN1A, TLR3, PI3Kd, AKT3, and PTEN were seen in the better responding group [78]. Finally, when we examined the ability of Ad5-based gene therapy vectors to infect canine lymphomas, we demonstrated that in addition to having reduced Coxsackie and Adenovirus Receptor (CAR), these cells also had reduced cell-surface integrins [79]. These findings indicated the importance of understanding the expression of multiple genes when looking at treatment possibilities.

### 3.5. Canine Transmissible Venereal Tumor—cTVT

Canine Transmissible Venereal Tumor (cTVT) is a venereal cancer of dogs most common in the developing world but found world-wide especially among feral and free-ranging domestic dogs. It is not a model of any human disease but appears to be the oldest known tumor in a small family of rare transmissible tumors that are transmitted through direct contact, do not involve a viral etiology and may well represent a unique system in which to investigate cancer immune suppression [80,81,82]. This includes the transmissible facial tumor of Tasmanian Devils as well as transmissible tumors in Syrian Hamsters and clams [81]. Such canine tumors are extraordinary as they have been calculated to have been circulating among wolves and feral dogs for at least 11,000 years and perhaps up to 17,000 years and thus co-date or perhaps even predate the divergence of domestic dogs from wolves which, although controversial, dates to at least 17,000 years ago and possibly much earlier [3,83,84]. This is based on comparative genomic sequence analysis of different cTVT isolates from several sites world-wide. cTVT is thought to have originated through UV-induced mutation, possibly in a macrophage, and appears to be clonal in origin [85]. cTVT encodes defects in, like other canine cancers, p16/INK4A, or CDKN2A genes, as well as c-Myc and Erg genes [81]. Although apparently defective in apoptosis, cTVT cells do express p53, Bcl-2 and p63 genes and defects in at least p53 protein expression were observed [86,87]. The tumor cells are highly aneuploid and appear not to express surface MHC molecules and thus exist in an immune privileged environment [80,81,85].

It is not known how cTVT avoids immune recognition buts this characteristic is one of the principal reasons for interest. Understanding how cTVT avoids immune recognition may well shed light on how other tumors can be manipulated to become more recognizable to the immune system promoting more effective immunotherapies [87]. Immune suppressed animals are thought to be most susceptible to cTVT [80]. In most cases of cTVT, immunosuppression is temporary and tends to fail in around 80% of cases by 6–9 months when the disease resolves [88]. This leaves the patient animal immune to further cTVT infection.

The genetics of cTVT transformation are not well understood although different clades have been detected and the disease can affect other canids such as coyotes and gray wolves [80,81,85]. Also not well known is that there appears to exist a second variant of cTVT that is found in South America, particularly in free-ranging dogs in urban environments in Chile, that has a distinctly different phenotype when assessed by a Veterinary Pathologist [Dr. Javier Ojeda Oyarzun, Valdivia University, Valdivia, Chile, personal communication]. This variant has not been sequenced to date and its relationship to the common wide-spread TVT variant is unknown [81]. Another example of a dendritic cell/cancer cell hybrid fusion vaccine has been reported for cTVT [89]. The vaccine has been reported to substantially reduce the time to regression of these tumors through amplification of the adaptive immune response and NK cell cytotoxicity.

### 3.6. Prostate Cancer

Canine prostate is morphologically and functionally similar to human prostate and canine prostate cancers are derived from urothelial or ductal cells [90]. The growth of prostate cancer in both species is often androgen-independent suggesting that canine prostate cancer is similar to hormone-resistant human prostate cancer [90].

Recently, canine prostate has been investigated and viable cell lines developed [90]. A variety of genes have been investigated but expression of the PTEN, mdm2 and p53 genes have been altered in canine prostate cancer as has STAT3 and these tumors are androgen dependent [91,92,93]. There are also reports that, unlike canine melanoma, canine prostate may harbor BRAF mutations and may be sensitive to docetaxel treatment [94].

### 3.7. Can Cancer Incidence Be Modified in Individual Dogs?

A number of studies over the years have addressed the question of cancer susceptibility and its management through the application of caloric restriction and detected a statistically reproducible extension of lifespan in mammalian species comparable to that observed in otherwise short-lived model species [95,96]. However, in a 15-year investigation that followed 48 Labrador Retrievers from birth to death half were fed an ad lib diet and half were fed at 75% of the ad lib caloric intake [95]. The median life span of the caloric restricted animals was almost 2 years longer over a median lifespan of 11.2 years for an enhanced lifespan improvement of 15%. By 13.5 years into the study, 25% of the caloric restricted dogs were still alive while all of the ad lib fed animals were deceased. Further, the surviving dogs suffered from far fewer maladies associated with aging including a significant delay in cancer development. The conclusions were clear. A significant improvement in health and longevity of canine pets can be achieved by controlling caloric intake, and thus diet and weight, and this includes significantly delaying any development of cancer [95]. However, care should be taken not to assume direct causation in this case, as it may more accurately reflect that animals on a higher plane of nutrition may represent “fertile ground” in which tumors, when they do occur, find it easier to become established. It does seem possible, however, that nutrient restriction can impact cancer development and thus may also influence either mutation or its fixation in tissues.

A second example of cancer incidence modification can be seen in canine mammary adenocarcinoma. The incidence of this tumor in intact female dogs is nearly identical to the incidence in women, with an approximate 1 in 8 lifetime incidence. If a female dog undergoes ovariohysterectomy before the first estrus cycle, her lifetime risk drops to a nearly unmeasurable level. Ovariohysterectomy after four estrus cycles (approximately 2 years of age) has almost no effect on mammary adenocarcinoma incidence while spaying at intermediate time points show an intermediate level of risk [32]. Such data strongly suggests that oncogenic mutations, and the cells containing them, are likely sensitive to hormone-dependent tumor promotion.

## 4. Concluding Comments

It is clear that the natural history and genetic profiles of spontaneous cancers in dogs provide an extraordinary opportunity to investigate accelerated models of cancer in an intermediate sized mammal that shares our lived environment and thus much of our environmental cancer risk. The genetics and natural histories of these tumors are also similar to human disease in many instances, making their use as models even more appropriate. Because dogs live a much shorter period than humans and their tumors are proportionally more progressive, they also offer timely opportunities to develop and test new therapeutics, especially those targeting the key tumor suppressor genes such as p53 and INK4A/p16 as well as oncogenes such as the EGFR family that are found to be commonly defective in both human and canine cancers. Promising investigations of immunotherapies and cancer vaccines support an optimistic outlook for the treatment of cancer in both human and canine patients.

## Figures and Tables

**Table 1:** Common Canine and Human Cancer Genes.

Common Canine Cancer Genes	
**Oncogenes**	
**Epidermal Growth Factor Receptor Gene Family**	c-erbB-1/EGFR
	c-erbB-2/HER2
	c-erbB-3
	c-erbB-4
**Tumor Suppressor Genes**	
	INK4A/p16
	P14ARF
	p53

**Table 2:** BrCa Phenotypes for Human and Canine Breast/Mammary Tumor Cells.

Human and Canine Breast/Mammary Cancer Phenotype Definitions			
Phenotype	ER Estrogen Receptor Alpha	PR Progesterone Receptor	HER2 c-erbB-2
**Luminal A**	+	+	−
	−	+	−
	+	−	−
**Luminal B**	+	+	+
	−	+	+
	+	−	+
**HER2**	−	−	+
**Triple Negative**	−	−	−

Quantitative reverse transcriptase PCR (QrtPCR) assays were utilized throughout. Canine and human breast/mammary cancer phenotypes are highly similar. Some HER2+ and Luminal B canine mammary tumors are c-*erb*B-3+ or both c-*erb*B-3+ and c-*erb*B-4+ as well. Adapted from Luful Kabir et al. 2017 [14].

## Data Availability

No new data were created or analyzed in this study. Data sharing is not applicable to this article.

## Abbreviations

The following abbreviations are used in this manuscript:

SKA2	Spindle and kinetochore-associated complex subunit 2 gene
NEU1	Neuraminidase 1 gene
ATAC	Assay for transposase-accessible chromatin
TIL	Tumor infiltrating lymphocyte
KIT	Receptor tyrosine kinase
Flt3	fms-like tyrosine kinase 3
SYK	Spleen-associated tyrosine kinase
TIMP1	TIMP metallopeptidase inhibitor 1
RPS/L	Ribosomal protein small/large subunit
EDEM1	ER degradation enhancing alpha-mannosidase-like protein 1
PTK2B	Protein tyrosine kinase 2 beta
MMP9	Matrix metalloproteinase 9
JAK1	Janus kinase 1
PCTL	Peripheral T-cell lymphomas
GATA3	Trans-Acting T-Cell-Specific Transcription Factor GATA-3
PI3K	Phosphoinositol 3 kinase
Akt	AKT Serine/Threonine Kinase 1
mTOR	Mechanistic Target Of Rapamycin Kinase
CCL	Chemokine
ITK	Interleukin-2-inducible T-cell kinase
LAT	Linker for activation of T-cells
CREBBP	cAMP response element-binding protein–binding protein
PD	Programmed death receptor/ligand 1 or 2
Myc	Myc oncogene
Erg	Erythroblast transformation-specific/ETS-related gene
Bcl-2	BCL2 Apoptosis Regulator gene
BRAF	Member of the RAS/MAPK pathway
CAR	Coxsackie and Adenovirus Receptor
MHC	Major histocompatibility gene
TLR	Toll-like receptor
DLBCL	Diffuse large B-cell lymphoma
BRCA	Breast cancer gene 1 and 2
PAF1	Polymerase-associated factor 1 complex subunit gene
PSMG2	Proteasome assembly chaperone 2
NOB1	NIN1/RPN12 binding protein 1 homolog gene
MDM2	Homolog of mouse double minute 2 p53-binding protein
PTEN	Phosphatase and tensin homolog gene
STAT3	Signal transducer and activator of transcription 3 gene
ER	Estrogen receptor
PR	Progesterone receptor
erb	EGF receptor B (epidermal-like growth factor)
HER-2	Human epidermal growth factor receptor 2
INK	Inhibitor of cyclin-dependent kinase
CDK	Cyclin-dependent kinase
ARF	Alternative reading frame
Q-RT-PCR	Quantitative reverse-transcriptase polymerase chain reaction
FISH	Fluorescence in situ hybridization
FHIT	Fragile histidine triad
CAR	Coxsakie and adenovirus receptor
CHOP	Cyclophosphamide, hydroxydaunorubicin, oncovin and prednisone therapy
cTVT	Canine transmissible venereal tumor
NK	Natural killer
